# Delayed Diagnosis and Diagnostic Pathway of ALS Patients in Portugal: Where Can We Improve?

**DOI:** 10.3389/fneur.2021.761355

**Published:** 2021-10-27

**Authors:** Catarina Falcão de Campos, Marta Gromicho, Hilmi Uysal, Julian Grosskreutz, Magdalena Kuzma-Kozakiewicz, Miguel Oliveira Santos, Susana Pinto, Susanne Petri, Michael Swash, Mamede de Carvalho

**Affiliations:** ^1^Institute of Physiology, Institute of Molecular Medicine, Faculty of Medicine, University of Lisbon, Lisbon, Portugal; ^2^Department of Neurosciences and Mental Health, Northern Lisbon University Hospital Centre, Lisbon, Portugal; ^3^Department of Neurology and Clinical Neurophysiology, Faculty of Medicine, Akdeniz University, Antalya, Turkey; ^4^Hans Berger Department of Neurology, Jena University Hospital, Jena, Germany; ^5^Department of Neurology, Neurodegenerative Disease Research Group, Medical University of Warsaw, Warsaw, Poland; ^6^Department of Neurology, Hannover Medical School, Hannover, Germany; ^7^Departments of Neurology and Neuroscience, Barts and the London School of Medicine, Queen Mary University of London, London, United Kingdom

**Keywords:** amyotrophic lateral sclerosis (ALS), diagnostic delay, time to diagnosis, diagnostic pathway, motor neuron disease

## Abstract

**Background:** Amyotrophic lateral sclerosis (ALS) is a progressive neurodegenerative disease with unsatisfactory treatment options. Best management and recruitment into clinical trials requires early diagnosis. However, diagnosis is often delayed. Analysis of the diagnostic pathway and identification of the causes of diagnostic delay are imperative.

**Methods:** We studied a cohort of 580 ALS patients followed up in our ALS clinic in Lisbon. Demographic, disease, and sociocultural factors were collected. Time from first symptom onset to diagnosis, the specialist's assessment, and investigations requested were analyzed. Predictors of diagnostic delay were evaluated by multivariate linear regression, adjusting for potential confounders.

**Results:** The median diagnostic delay from first symptom onset was 10 months. Spinal-onset, slower disease progression, cognitive symptoms at onset, and lower income were associated with increased diagnostic delay. Most patients were first assessed by general practitioners. Patients who were first evaluated by a neurologist were more likely to be correctly diagnosed, decreasing time to diagnosis. Electromyography was decisive in establishing the diagnosis.

**Conclusions:** Late referral from non-neurologists to a neurologist is a potentially modifiable factor contributing to significant diagnostic delay. Educational interventions targeted to non-neurologists physicians, in order to increase awareness of ALS and, consequently, promote early referral to a neurologist at a tertiary center, will be important in reducing diagnostic delay.

## Introduction

Amyotrophic lateral sclerosis (ALS) is characterized by progressive loss of motor neurons in the brain and spinal cord, leading to muscle weakness and death due to respiratory failure (1). Despite the rapidly progressive course, the median time to diagnosis of ALS patients is ~12 months, which is not adequate, since the median life expectancy is only 3 years (2, 3). Diagnostic delay may be linked to both disease phenotype and healthcare factors, especially early referral to a neurologist experienced in neuromuscular disease (3). Prompt diagnosis is important for several reasons. First, although currently available disease modifying treatments have limited efficacy, such therapies are likely to be more efficacious in the early stages of disease (4). Second, management in specialized ALS clinics has a positive impact on survival and quality of life in ALS patients (5). Third, early diagnosis may be decisive for inclusion in clinical trials. Finally, diagnostic delay constitutes a significant burden for ALS patients and healthcare systems with unnecessary consultations and diagnostic tests and even inappropriate surgery, causing ongoing diagnostic and prognostic uncertainty (6). Therefore, analyzing the diagnostic pathway and recognizing the different factors associated with diagnostic delay are important.

## Materials and Methods

### Study Population

We studied adult Portuguese ALS patients followed up from January 2015 to January 2018 in our ALS clinic in Lisbon, who agreed to the study. We included patients with definite, probable, probable laboratory-supported, and possible ALS, according to the revised El-Escorial criteria. Patients with progressive muscular atrophy (PMA) were also included, as PMA is accepted as a phenotype of ALS (7), but we excluded 46 patients with monomelic motor neuron disease, Kennedy disease, and primary lateral sclerosis since those disorders have a different pattern of progression. Moreover, we excluded patients unable to provide reliable information regarding their diagnostic track, even with the caregivers' contribution.

### Data Collection

Demographic and clinical data were obtained at the first visit to the ALS clinic by strictly applying a standardized questionnaire developed in the OnWebDuals project and published elsewhere (8). Place of living (rural vs. urban areas) and main occupations before disease onset, classified according to the International Standard Classification of Occupations (ISCO), were also collected. The average monthly income was estimated using the Eurostat data (European Commission—https://ec.europa.eu/eurostat/data/database).

Diagnostic delay was estimated from symptom onset to diagnosis. To understand the diagnostic pathway of ALS patients, data regarding number of medical observations, medical specialists involved (neurologist vs. non-neurologist) until diagnosis, time from first symptoms to first medical observation, time from the first medical to the second medical observation, and investigations requested (including CT or MR imaging and neurophysiological studies) were analyzed. The diagnostic pathway was further explored in three subgroups of ALS patients classified by region of onset: bulbar-onset, upper limb, and lower limb spinal-onset. To analyze the impact of the rate of functional decline on diagnostic delay, the ALS Functional Rating Scale—Revised (ALSFRS-R) decline rate was calculated (48-ALSFRS-R at study entry/number of months since first symptoms).

The project was approved by the local ethics commission. All patients gave written informed consent before inclusion in the study.

### Statistical Analysis

Data analysis was performed with STATA 13 software. For descriptive analysis, means, medians, standard deviations, and interquartile ranges were calculated for continuous variables and percentages for categorical variables. Predictors of diagnostic delay were identified using uni- and multivariate linear regression models. Predictors strongly associated with the outcome in univariate models were included in the final model. Student's *t*-test or Mann–Whitney *U*-test were used to compare continuous variables and the Chi-squared test to compare categorical data between patients with neurological and non-neurological assessments. A *p*-value < 0.05 was considered statistically significant.

## Results

From the initial 580 patients, we excluded 1 patient for missing diagnostic date. Most patients were classified as probable ALS according to the El Escorial criteria, and 22% of patients were diagnosed with PMA. Approximately one-fifth of patients had a bulbar-onset (22%). The baseline characteristics are detailed in Table 1.

**Table 1:** Baseline characteristics of ALS patients.

**Baseline characteristics (***n*** = 580)**	
Age (years)	65 ± 12 (23–89)
Gender (male)	335 (58%)
**El Escorial criteria**	
Definite	102 (18%)
Probable	266 (46%)
Possible	25 (4%)
Probable-laboratory supported	59 (10%)
**Alternative diagnosis**	
PMA	127 (22%)
**UMN vs. LMN at onset**	
UMN	178 (31%)
LMN	390 (68%)
UMN and LMN	8 (1%)
Bulbar-onset	128 (22%)
Respiratory onset	22 (9%)
ALSFRS-R rate of decline (per month)	0.9 ± 1.1 (0-12)
Cognitive symptoms at onset	29 (5%)
ALS/FTD family history	57 (10%)
**Place of living**	
Rural area	84 (15%)
Urban area	491 (85%)
**Monthly average income (euros)**	
<1,000	328 (57%)
>1,000	235 (40%)

*ALS, amyotrophic lateral sclerosis; ALSFRS-R, ALS Functional Rating Scale—Revised; FTD, frontotemporal dementia; LMN, lower motor neuron; PMA, progressive muscular atrophy; UMN, upper motor neuron*.

**Figure 1:**
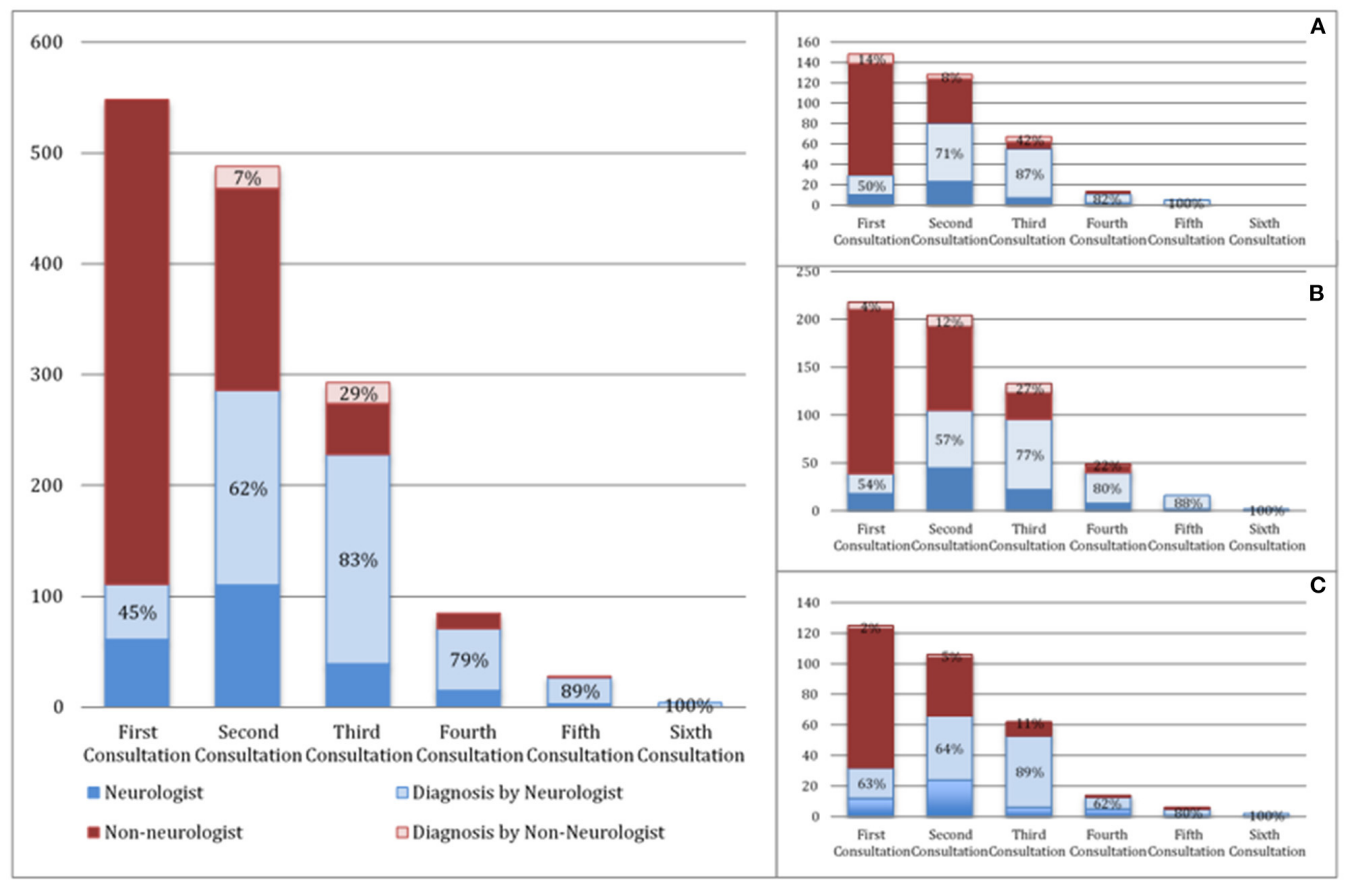
Diagnostic pathway of ALS patients (**A**—upper limbs onset, **B**—lower limbs onset, **C**—bulbar-onset). The proportion of neurologists (light blue) and non-neurologists (light red) who made the diagnosis is represented in percentages.

**Figure 2:**
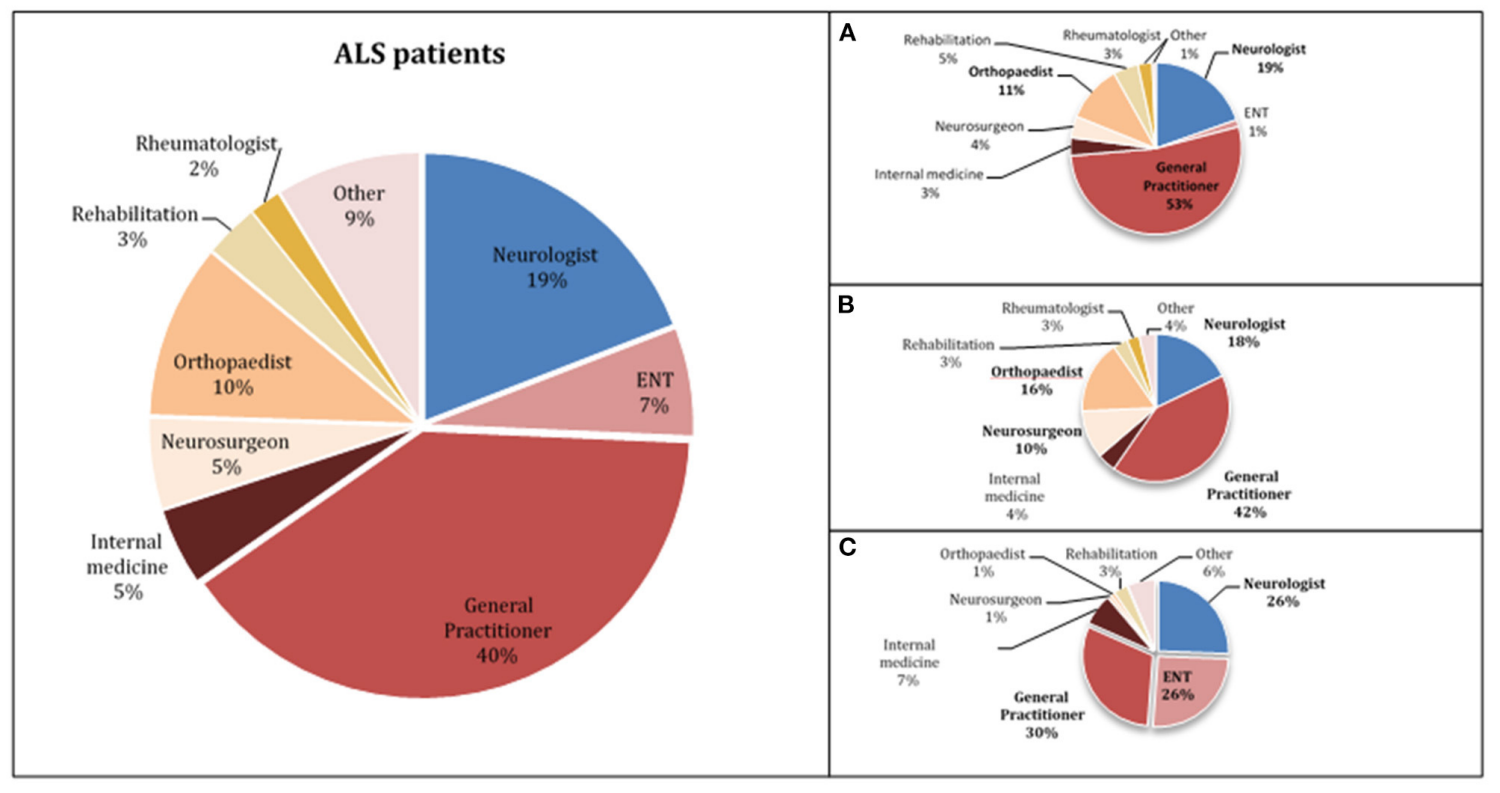
Specialist physicians who first evaluated ALS patients (**A**—upper limbs onset, **B**—lower limbs onset, **C**—bulbar-onset).

In our population, the median diagnostic delay from first symptom was 10 months (1st−3rd IQR = 5–18). In the multivariate linear regression analysis (Table 2), patients with bulbar-onset and faster disease progression (higher ALSFRS-R rate of decline) had a shorter diagnostic delay (coef. **–**9.61, *p* < 0.001; coef. **–**5.75, *p* < 0.001, respectively). Cognitive symptoms at onset were associated with a longer diagnostic delay (coef. 10.08, *p* = 0.016), as well as a lower monthly income (coef. 3.67, *p* = 0.046). Living in a rural or urban area was not a predictor of diagnostic delay. Moreover, age, gender, predominant upper (UMN) or lower motor neuron (LMN) presentation, diaphragm onset, and family history of ALS/frontotemporal dementia (FTD) were not predictors for the diagnostic delay. The median diagnostic delay was also not influenced by known comorbidities such as previous spinal surgery, stroke, or diabetes. The median time from first medical consultation to diagnosis was 6 months (1st−3rd IQR = 5–18). The majority (70%) of patients had two or three medical evaluations until diagnosis, and almost 15% consulted at least four specialists. The diagnostic pathway is represented in Figure 1. Most patients were first evaluated by a non-neurologist (80%), usually a general practitioner (GP), and only 20% consulted a neurologist first (Figures 1, 2). In patients first seen by a neurologist, the diagnosis of ALS was established in 45%, but 55% of the patients went to a second neurologist before receiving the correct final diagnosis (Table 3). Additionally, in patients who were initially assessed by a neurologist, the time to diagnosis was shorter than in patients first evaluated by a non-neurologist (3 vs. 6 months, respectively; *p* < 0.001). However, patients took a longer time to be first assessed by a neurologist than by non-neurologists (4 vs. 2 months, respectively; *p* = 0.01). No differences regarding age, gender, region at onset, and disease progression were found between both groups (neurologist vs. non-neurologist at first appointment). Diagnostic delay was similar between ALS and PMA patients [10 months (1st−3rd IQR = 5–18) vs. 10 months (1st−3rd IQR = 6–22, respectively, *p* = 0.55)]. Only 14% of patients with PMA first consulted a neurologist, even lower than ALS patients (14 vs. 24%; *p* = 0.04). In our cohort of patients, 22 patients had respiratory-onset ALS. The median diagnostic delay was 7.5 months (1st−3rd IQR = 4.0–11.1), lower than the remaining population. However, in the multivariate analysis, respiratory onset was not significantly associated to a lower diagnosis delay (Table 2). Only two patients (9%) were first assessed by a neurologist, and the remaining patients were mainly evaluated by a GP, cardiologist, or pneumologist.

**Table 2:** Multivariate linear regression analysis assessing predictors of diagnostic delay in ALS patients.

	**Coefficient (95% CI)**	***P*** **-value**
Age (years)	−0.05 (−0.20, 0.10)	0.537
Gender (male)	−1.00 (−4.61, 2.61)	0.585
El Escorial criteria	−0.39 (−2.42, −1.65)	0.708
**Bulbar-onset**	**−9.61 (−14.30**, **−4.92)**	**<0.001**
**ALSFRS-R rate of decline (per month)**	**−5.75 (−7.42**, **−4.08)**	**<0.001**
UMN manifestation at onset	8.10 (−4.59, 20.81)	0.210
LMN manifestation at onset	4.24 (−8.51, 16.99)	0.514
Respiratory onset	−8.51 (−21.13, 4.11)	0.186
**Cognitive symptoms at onset**	**10.08 (1.88-18.29)**	**0.016**
ALS/FTD family history	−0.42 (−6.06, 5.22)	0.885
Place of living (urban area)	0.54 (−4.42, 5.50)	0.831
**Monthly average income (<1,000 euros)**	**3.67 (0.06, 7.28)**	**0.046**

*ALS, amyotrophic lateral sclerosis; ALSFRS-R, ALS Functional Rating Scale—Revised; FTD, frontotemporal dementia; LMN, lower motor neuron; UMN, upper motor neuron*.

*Bold values highlight the predictors of diagnosis delay which were statistically significant (p < 0.05)*.

**Table 3:** Comparison between ALS patients who were first observed by a neurologist and non-neurologist.

	**Neurologist (***n*** = 111)**	**Non-neurologist (***n*** = 436)**	***p*** **-value**
Age (years)	66.1 ± 1.2	64.7 ± 0.6	0.28^**^
Gender (male)	62%	56%	0.26^***^
Spinal-onset	59%	69%	0.05^***^
Bulbar-onset	29%	21%	0.09^***^
ALSFRS-R rate of decline (per month)	0.6 (0.2–1.5)	0.5 (0.3–1.0)	0.55^***^
**Median time between symptom onset and first medical observation (months, 1st−3rd IQR)**	**4 (1–7)**	**2 (1–10)**	0.01^***^
**Median time between first medical observation and diagnosis (months, 1st−3rd IQR)**	**3 (1–8)**	**6 (3–13)**	<0.001^***^
**Investigation requested**			
EMG	**75%**	**20%**	<0.001^**^
Brain CT scan	21%	0.1%	<0.001^***^
Cervical CT scan	0.05%	0.04%	0.93^***^
Lumbo-sacral CT scan	0.01%	0.1%	0.005^***^
Brain MRI	36%	0.05%	<0.001^***^
Cervical MRI	42%	0.07%	<0.001^***^
Thoracic MRI	0.2%	0.02%	<0.001^***^
Lumbo-sacral MRI	0.2%	0.1%	0.091^***^
**Made the diagnosis**	**45%**	**0%**	<0.001^**^
Time to second medical observation (months)	4 (2–7.5)	3 (1–6)	0.12^***^

*ALS, amyotrophic lateral sclerosis; ALSFRS-R, ALS Functional Rating Scale—Revised; EMG, electromyography; CT, computed tomography; MRI, magnetic resonance imaging*.

* *Student's t-test*.

** *Chi-squared-test*.

*** *Mann–Whitney U-test*.

*Bold values highlight the differences that are statistically significant*.

Taking into account the wide spectrum of clinical presentation in ALS patients, we further analyzed the diagnostic pathway in different subgroups of patients, particularly patients with bulbar and spinal onset (upper limbs—UL and lower limbs—LL) as illustrated in Figures 1, 2. As expected, bulbar-onset patients were frequently referred to an otorhinolaryngologist (ENT). In spinal-onset patients, first referral to a neurosurgeon or orthopedic surgeon was common (Figures 1, 2). In all groups, the odds of ALS diagnosis increased for those patients who were afterwards referred to a neurologist. Almost all specialists who made the diagnosis (95%) requested an electromyographic (EMG) investigation. Other investigations were also requested, including brain and cervical MRI in ~25% of patients.

## Discussion

The median diagnostic delay was 10 months in our patient cohort. Most studies with cohorts of ALS patients reported from different countries, and with distinct health systems, have reported similar findings with a delay of 10–16 months (3). As previously and consistently described, bulbar-onset patients had a shorter time to diagnosis (9–13). Although patients with bulbar-onset progress more rapidly than spinal-onset patients, a faster rate of disease progression was independently associated with a shorter diagnostic delay (6, 13). Other unmodifiable factors such as age, gender, and predominant UMN/LMN presentation have been studied as potential predictors of diagnostic delay, with variable results (6–14). In our study, none of these were independent factors for diagnostic delay. These discrepancies among previous studies may be related to different potential confounders, such as disease progression rate, that may have not been assessed. We also found that the presence of cognitive symptoms at onset was associated with a longer diagnostic delay. To our knowledge, solely two previous studies described the possible impact of cognitive defect in the diagnosis delay in ALS patients. Nonetheless, no conclusions could be drawn since a very small number of patients were included (12, 15). Family history of ALS/FTD has also been addressed in two previous studies, suggesting a shorter diagnostic delay (6, 11). However, our study does not support this finding. Since familial ALS represents only 10% of patients, a statistically significant association may be difficult to achieve, requiring much larger cohorts of ALS patients.

Considering that time between symptom onset and first medical assessment is inevitably correlated with diagnostic delay, factors that influence access to healthcare, such as place of living and educational level or income, may be of interest (6, 12, 16). Place of living (rural vs. urban areas) was not associated with diagnostic delay in our study; however, lower income was an independent factor for diagnosis delay. This finding may be related to two main aspects. First, lower income is generally related to a lower level of education, which may contribute to difficulty in patients recognizing motor symptoms requiring specialized medical care. Second, in Portugal, patients with lower income usually have access only to the public healthcare system. Consequently, patients are initially consulted by a GP in primary health centers and then referred to a specialist, whereas patients with higher income benefit from private health insurance, facilitating more rapid access to a specialized medical care, e.g., a neurologist.

The median time from first medical observation to diagnosis was 6 months, slightly higher than reported by Paganoni et al. (11). We believe that this period is far from ideal and should be further reduced. Since the great majority of patients were first assessed by a non-neurologist, namely, a GP, educational interventions should take place in primary healthcare centers in order to promote increased awareness of ALS by the GPs, allowing prompt referral to a neurologist. Patients who were evaluated by a neurologist had increased odds of being diagnosed with ALS, as expected. Nonetheless, not all neurologists correctly diagnosed ALS patients at first evaluation (55%), even if an EMG was requested (75%). Although not systematically investigated, the most common alternative diagnosis in the EMG report was severe root lesion and spinal stenosis, in particular for patients with LL onset and preserved UL, bulbar, and respiratory function.

EMG findings, serving as surrogate of LMN degeneration, were included in the Awaji criteria, increasing the sensitivity of ALS diagnosis (17, 18). Several studies further proved the relevance of neurophysiological studies in the diagnosis of ALS (12, 19, 20), and in our cohort, almost all specialists who made the diagnosis at some point requested an EMG. However, neurophysiological tests are best performed in the context of a clinical suspicion. We are optimistic that the simpler Gold Coast criteria could facilitate ALS diagnosis (7). However, it is likely that only the availability of effective treatment will really change medical behavior.

Our study has some limitations. First, considering the prognosis of ALS patients, some physicians may be restrained and inclined to diagnose more benign and treatable conditions, increasing the diagnosis delay. Nonetheless, in our study, most patients were diagnosed in our center (80%) in which we prefer to give a diagnosis when established than deferring it by asking for more non-relevant investigations. However, this could be a problem in other centers concerning patients with a previous diagnosis, although this is not a general practice in Portugal. Second, the alternative diagnosis established before the ALS diagnosis, contributing to a diagnostic delay, was not systematically evaluated. However, as previously described, the most common misdiagnosis in ALS patients has been radiculopathy, spinal cord lesions, rheumatologic disorder, stroke, and myasthenia gravis (for bulbar-onset patients) and less frequently myopathy, motor neuropathies, and other neurodegenerative disorders (11, 12).

Our study is original in considering socioeconomic factors as influencing the diagnostic pathway in ALS patients. Our findings acknowledge that ALS diagnosis is still significantly delayed. We acknowledge that the time to diagnosis has remained unchanged over the years and is similar in distinct national healthcare systems (3), which raises doubts regarding the universal importance of socioeconomic factors on diagnostic delay. However, a late referral to a neurologist has been shown to be a significant and potentially modifiable factor associated with diagnostic delay. In this regard, more rapid referral to the neurologist is probably relevant. No simple and sensitive biomarker of ALS is available. Therefore, promotion of educational programs targeted to GPs, or other medical specialists who may evaluate ALS patients (e.g., neurosurgeons, orthopedists, ENT specialists), in order to increase awareness of ALS, and the need for urgent referral to a neuromuscular center may be a paramount strategy to decrease time to diagnosis.

## Data Availability Statement

All analyses and anonymized data will be shared by request from any qualified investigator and after consideration of the scientific project.

## Ethics Statement

The studies involving human participants were reviewed and approved by Comissão de Ética do Centro Académico de Medicina de LIsboa - CAML. The patients/participants provided their written informed consent to participate in this study.

## Author Contributions

CF: analysis and interpretation of data and writing the draft. MG: data collection, interpretation of data, and review of the manuscript. HU, JG, MK-K, SPi, SPe, and MS: review of the draft. MO: data collection and review of the draft. MC: study conception, data collection, interpretation of the data, and review of the draft. All authors reviewed the results and approved the final version of the manuscript.

## Funding

Project Brainteaser—Bringing Artificial Intelligence home for a better care of amyotrophic lateral sclerosis and multiple sclerosis funded by the European Union' Horizon 2020 research and innovation program under Grant Agreement No. GA101017598.

## Conflict of Interest

The authors declare that the research was conducted in the absence of any commercial or financial relationships that could be construed as a potential conflict of interest.

## Publisher's Note

All claims expressed in this article are solely those of the authors and do not necessarily represent those of their affiliated organizations, or those of the publisher, the editors and the reviewers. Any product that may be evaluated in this article, or claim that may be made by its manufacturer, is not guaranteed or endorsed by the publisher.
